# Impact of a Pressure Injury Prevention Bundle in the Solutions for Patient Safety Network

**DOI:** 10.1097/pq9.0000000000000013

**Published:** 2017-02-16

**Authors:** Gary Frank, Kathleen E. Walsh, Sharyl Wooton, Jim Bost, Wei Dong, Leah Keller, Michelle Miller, Karen Zieker, Richard J. Brilli

**Affiliations:** From the * Children’s Healthcare of Atlanta Children’s Hospital of Atlanta, Atlanta, Georgia; † Department of Pediatrics, Emory University School of Medicine, Atlanta, Georgia; ‡ James M. Anderson Center for Health Systems Excellence, Cincinnati Children’s Hospital Medical Center, Cincinnati, Ohio; § Liberty Mutual Insurance, Boston, Mass.; ¶ Hospital Administration, Department of Quality Improvement, Department of Pediatrics, Division of Critical Care Medicine, Nationwide Children’s Hospital, Columbus, Ohio; and ‖ The Ohio State University College of Medicine, Columbus, Ohio.

## Abstract

**Background:**

Our objective was to describe changes in pressure injury (PI) rates in pediatric hospitals after implementation of an active surveillance and prevention bundle and to assess the impact of bundle elements.

**Methods:**

The Children’s Hospitals Solutions for Patient Safety (SPS) Network is a learning collaborative working together to eliminate harm to hospitalized children. SPS used a 3-pronged approach to prevent pressure injuries: (1) active surveillance, (2) implementing and measuring compliance with the prevention bundle, and (3) deploying a wound ostomy team. Among hospitals participating since 2011 (phase 1), we used negative binomial analyses to assess change in PI rates. Only phase 1 hospitals had a baseline period before any prevention bundle intervention. Among all hospitals participating in 2013 (phases 1 and 2), we used funnel charts to assess the association between reliable bundle implementation and PI rates.

**Results:**

Among the 33 hospitals that participated in SPS from 2011 to 2013 (phase 1), the rate of stage 3 pressure injuries declined from 0.06 to 0.03 per 1,000 patient-days (*P* < 0.001). Stage 4 pressure injuries declined from 0.01 to 0.004 per 1,000 patient-days (*P* = 0.02). Among all 78 hospitals in phases 1 and 2, the cohort that adopted each bundle element, measured compliance, and achieved 80% prevention bundle compliance had significantly lower PI rates compared with all hospitals.

**Conclusions:**

SPS hospitals saw a significant reduction in stage 3 and 4 PIs over a 2-year period. Reliable implementation of each element of a prevention bundle was associated with lower PI rates.

## Abstract 2

Supplemental Digital Content is available in the text.

## BACKGROUND

Hospital-acquired pressure injuries among children are increasingly recognized as a significant cause of morbidity and can add considerably to the cost of hospitalization.^1–5^ Pressure injuries occur in up to 27% of pediatric intensive care unit patients and up to 23% of neonatal intensive care unit patients.^6,7^ In a study of 9 US children’s hospitals, an overall pressure injury (PI) prevalence of 4% was noted.^7^ Treatment of a single PI might cost $70,000 in adults^2,8,9^ and approximately $20,000 in children.^10^

Several studies have evaluated PI risk factors in hospitalized children. Pressure injuries develop from tissue destruction due to pressure exerted on the tissue between a bony prominence and an external surface.^2,11^ In a national survey, participants cited sedation, hypotension, sepsis, spinal cord injury, traction devices, and terminal illness as risk factors for PI development.^12^ In a study of patients admitted to a pediatric intensive care unit, risk factors included the presence of edema, prolonged length of stay, high positive-end expiratory pressure in ventilated patients failing to turn the patient, and weight loss.^13^ Also, medical devices such as oxygen saturation probes, intravenous securement devices, BiPAP masks, endotracheal and tracheotomy tubes, catheters, and splints are being increasingly recognized as causes of pressure injuries in children.^7^

Hospitals are facing increased demand to prevent hospital-acquired pressure injuries. The National Quality Forum considers any stage 3, stage 4, or unstageable PI acquired after admission a “Serious Reportable Events.”^14^ Additionally, the Center for Medicare and Medicaid Services considers stage 3 and 4 pressure injuries, not present on admission as “hospital-acquired conditions” for which hospitals can no longer bill additional charges.^15^ Although these rules initially applied to Medicare patients, many Medicaid and private insurers are now following suit.

Using care bundles to avoid hospital-acquired conditions is a quality improvement methodology that is gaining increasing recognition.^16–19^ A bundle is “a set of interventions, preferably evidence-based, intended for a defined patient population and care setting that, when implemented together, will result in better outcomes than when implemented individually.”^20^ Typically, a bundle has 3–5 relatively independent elements that are accepted by clinicians as care that should be delivered as usual practice. Bundle reliability is defined as the percentage completion of all items of the bundle. High reliability is a critical foundational tenant of safe healthcare. According to Weick and Sutcliffe,^21^ processes that lead to high reliability include preoccupation with failure, reluctance to simplify explanations, sensitivity to operations, commitment to resilience, and deference to expertise.

There is an abundance of literature regarding PI prevention strategies in adults; however, the pediatric literature is limited.^1,2,5^ Furthermore, there is no standard pediatric prevention bundle focused on pressure injuries. This report describes the work of a collaborative designed to improve detection and prevention of hospital-acquired pressure injuries in children. The Children’s Hospitals Solutions for Patient Safety (SPS) Network, a network of more than 100 children’s hospitals working together to eliminate serious harm to hospitalized children, developed and tested an approach to pediatric PI prevention. For PI, the network goal was to reduce the number of serious pressure injuries, defined as stage 3, stage 4, unstageable pressure injuries, and deep tissue injuries. The objective of this article is to describe changes in PI rates in pediatric hospitals after implementation of an active surveillance PI detection process and evidenced-based prevention bundle and to assess the impact of bundle elements on rates of PI.

## METHODS

### Setting

The Children’s Hospitals SPS Learning Network is one of the hospital engagement networks created as part of the Center for Medicare and Medicaid Services Partnership for Patients.^22^ Each participating institution in SPS is committed to the learning network guiding principles, rooted in the Institute for Healthcare Improvement’s Breakthrough Series Approach.^23^ The Institute for Healthcare’s approach includes (1) outcomes transparency; (2) data sharing; (3) “all teach, all learn” to promote sharing and learning from both successes and failures in implementing recommended strategies; and (4) commitment from participating senior hospital leadership to avoid interinstitutional competition on safety. The national network started in 2011 with 33 hospitals (phase 1); it grew to 78 hospitals (phase 2) by 2013 and currently has over 100 hospitals.^24^ These hospitals account for approximately 50% of admissions to children’s hospitals in the United States and nearly 25% of all pediatric admissions in the United States. The Institutional Review Board at Cincinnati Children’s Hospital, the lead site, reviewed the SPS collaborative work and considered it exempt.

The network (phase 1 hospitals) collected baseline data from January to December 2011. Throughout 2012, the network implemented improvements. We considered this time a ramp-up period to facilitate communication of requirements and to allow hospitals to organize improvement processes. Phase 2 hospitals, because they were joining a network actively engaged in the prevention of pressure injuries, did not use a baseline period.

### PI Prevention Bundle

SPS recommended a 5-element PI prevention bundle (Table 1) based on a pediatric literature review and a survey of the initial group of hospitals that reported lower PI rates. The recommended bundle was released in September 2012. Hospitals were asked to use a risk assessment tool of their choice for all patients and to apply the bundle to all high-risk patients, regardless of age, location in the hospital, or diagnosis. We defined bundle compliance as completion of all 5 components of the bundle. Hospitals measured bundle compliance either by direct observation or by documentation of the bundle elements in the medical record. Bundle reliability was defined as the percentage of patients in which participating hospitals competed full bundle compliance (ie, all 5 bundle components).

**Table 1.**
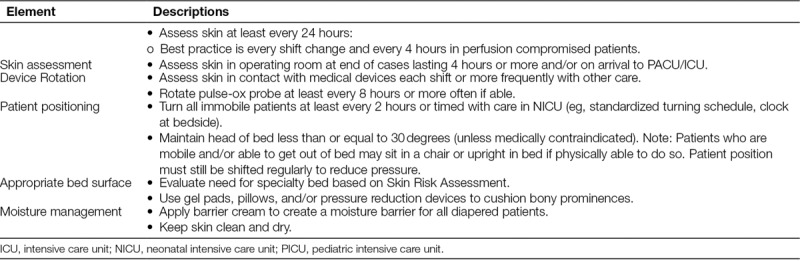
Recommended PI Bundle

The SPS network recommended a 3-pronged approach to the identification and prevention of pressure injuries: (1) conduct active surveillance to detect pressure injuries, (2) implement and measure compliance with the PI prevention bundle, and (3) deploy a wound ostomy team. We defined active surveillance as the periodic (best practice is weekly but at a minimum monthly) full skin assessment of every patient in a particular unit by a team including at least a Wound Ostomy Certified nurse, bedside nurse, and unit PI nurse champion. A Wound Ostomy Certified nurse is a registered nurse who has been formally trained to provide care for stomas, wounds, fistulas, drains, pressure injuries, and/or continence disorders.

### Measures

The SPS network provides each hospital a document outlining the operational definitions, data collection requirements, and calculations utilized by the network. Hospitals reported cumulative numbers of stage 2, stage 3, stage 4, unstageable, and deep tissue pressure injuries, along with total hospital patient-days (inpatients or observation patients) by month. For this analysis, our primary outcome metrics were severe pressure injuries, including stage 3, stage 4, deep tissue injuries, and unstageable pressure injuries. We removed hospitals with any missing data from the data set for that month.

### Survey

In March 2014, the SPS lead for each phase 1 and 2 hospital received an email survey. Survey questions included the following: (1) Which elements of the recommended prevention bundle did the hospital implement? (2) What additional practices did the hospital had to prevent PI? (3) What was the compliance for each of the SPS PI bundle elements during the period from July 2013 to December of 2013? (4) To which hospital units does the bundle compliance data belong? Hospitals received 3 email reminders and targeted phone calls to improve response rates.

### Analysis

Our primary outcome was the rate of severe pressure injuries. We calculated the rate of severe PI as the total number of stage 3, stage 4, deep tissue, and unstageable pressure injuries reported by all hospitals divided by the aggregate number of all hospital patient-days. We used control charts to plot the monthly PI rate. SPS defined the rule for centerline shifts *a priori* (ie, after 8 points above or below the centerline).^25^ Analysis was performed at the hospital level. Hospitals entered data on a monthly basis.

### Change in Rate of PI over Time (Phase 1 Hospitals Only)

We calculated PI rates for stage 2, 3, 4, and deep tissue pressure injuries during 3 study periods: baseline (2011), implementation period (2012), and study period (2013). The analysis was performed on data from the 33 hospitals that joined the network in 2011 because only those hospitals participated in the baseline period. We calculated mean rates for each PI category by dividing the total number of pressure injuries reported by all hospitals during that period by the aggregate number of hospital patient-days. Mean rates between the baseline and study periods were compared via 2-sample proportion tests with continuity correction.

We built adjusted analyses using random effects negative binomial regression models to relate the rates of different types of pressure injuries to month and study period. We selected the negative binomial regression model due to the large number of 0 values and resultant overdispersion in the distribution. We compared PI rates during the implementation and study periods with the baseline period. Incidence rate ratios and rate changes were computed to show the trend of rate changes controlling for the month and accounting for the variability of individual hospitals. Statistical analysis was performed using Stata 13.1 (StataCorp LP.). The *menbreg* package was used for mixed effects negative binomial models.^26^

### Analysis of Bundle Element Survey (Phases 1 and 2)

We analyzed the relationship between bundle compliance and PI outcomes on data from all hospitals that responded to the survey. For each bundle element, we divided hospitals into 4 categories: (1) hospitals that did not implement specific bundle elements; (2) hospitals that implemented bundle elements but did not measure bundle compliance (bundle reliability); (3) hospitals that implemented bundle elements, measured, and reported bundle reliability below 80%; and (4) hospitals that implemented bundle elements, measured, and reported bundle reliability^11^ above 80%. We selected 80% bundle reliability because this level of reliability suggests an association between process measures and outcomes.^16,18^

We used funnel chart analysis to demonstrate the relationship among these 4 categories of bundle implementation, each bundle element, and the rate of pressure injuries. The purpose of the funnel chart analysis was to demonstrate that each element of the bundle is independently associated with lower pressure ulcer rates and therefore should be included in the recommended bundle. We plotted funnel charts using both 95%ile and 97.5%ile control limits.^9,27^ The centerline represents the rate of events for all hospitals included in the analysis, as calculated by the sum of the number of events for each group divided by the sum of the number of patient-days for each group. The rates for each of the 4 groups were plotted using rate on the *y* axis and number of patient-days on the *x* axis. We used standard Shewhart u-chart methods to calculate upper and lower control limits.

## RESULTS

### Change in Rates of PI from Baseline after Intervention Period (Phase 1 Hospitals Only)

**Fig. 1.**
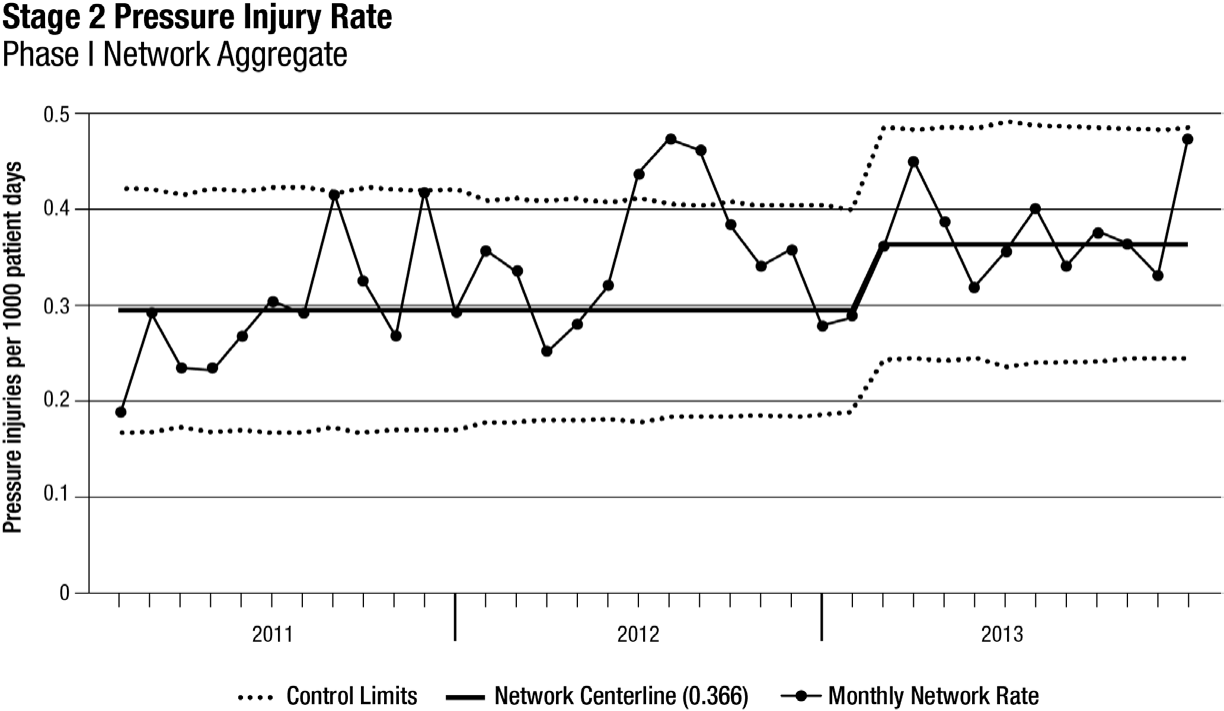
Stage 2 PI rates over time for phase 1 hospitals (n = 33).

**Fig. 2.**
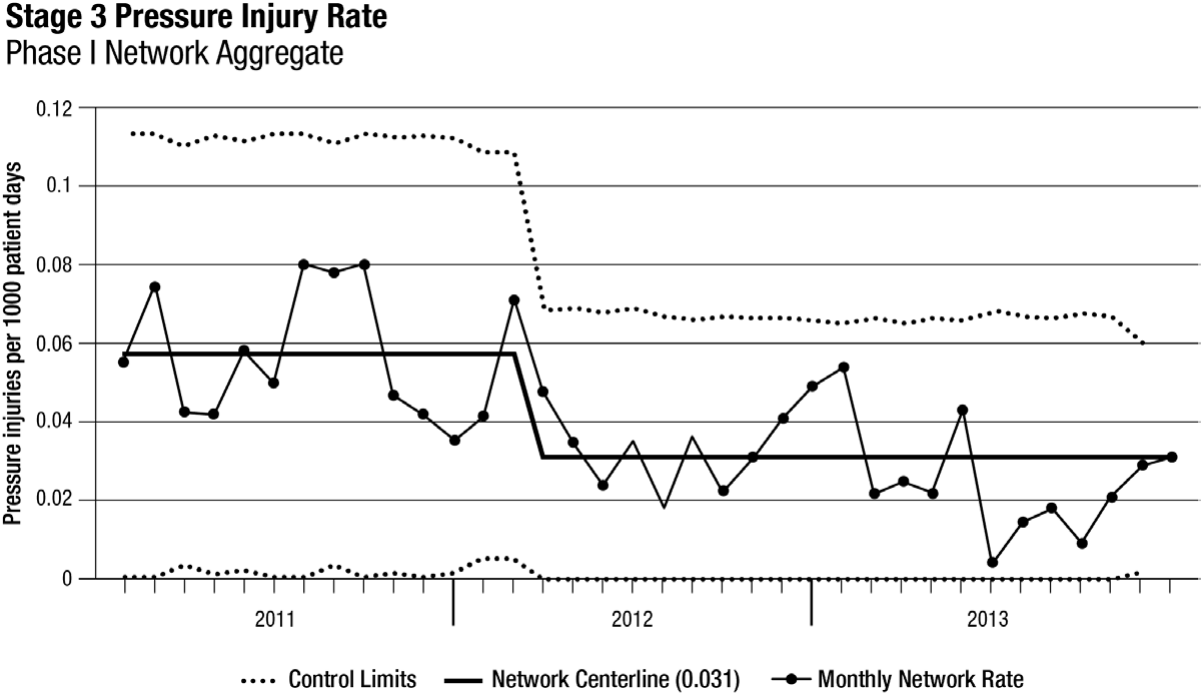
Stage 3 PI rates over time for phase 1 hospitals (n = 33).

**Fig. 3.**
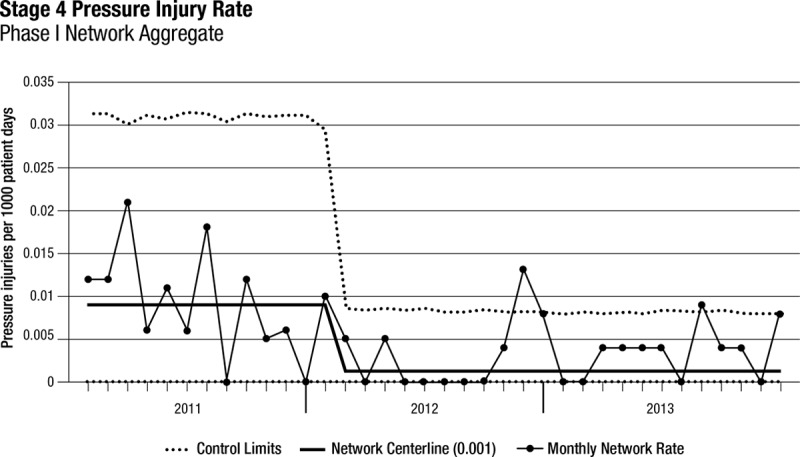
Stage 4 PI rates over time for phase 1 hospitals (n = 33).

Thirty-three freestanding children’s hospitals participated in SPS from January 1, 2011, until December 31, 2013 (phase 1). All 33 hospitals submitted at least 10 months per year of complete data, and 22 hospitals provided complete data throughout the 3-year period. Among these 33 hospitals, the rate of stage 2 pressure injuries increased from 0.3 to 0.38 per 1,000 patient-days (*P* < 0.001; Table 2, Fig. 1). The rate of stage 3 pressure injuries decreased from 0.06 to 0.03 per 1,000 patient-days (*P* < 0.001; Table 2, Fig. 2). The rate of stage 4 pressure injuries declined from 0.01 to 0.004 per 1,000 patient-days (*P* = 0.02; Table 2, Fig. 3). The rate of deep tissue injuries increased from 0.11 to 0.15 per 1,000 patient-days (*P* < 0.001; Table 2). The rate of unstageable pressure injuries rose from 0.08 to 0.09. However, this was not statistically significant (*P* = 0.18; Table 2). Finally, the aggregate rate of stage 3, stage 4, deep tissue injuries, and unstageable pressure injuries was not significantly different between the baseline and study periods.

**Table 2.**
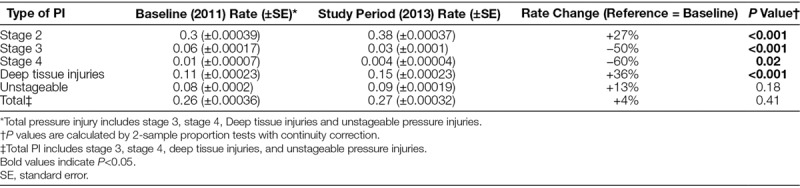
Rate per 1,000 Days of Pressure Injuries During Baseline and Study Periods for First 33 Hospitals in the Network (Phase 1 Hospitals)

**Fig. 4.**
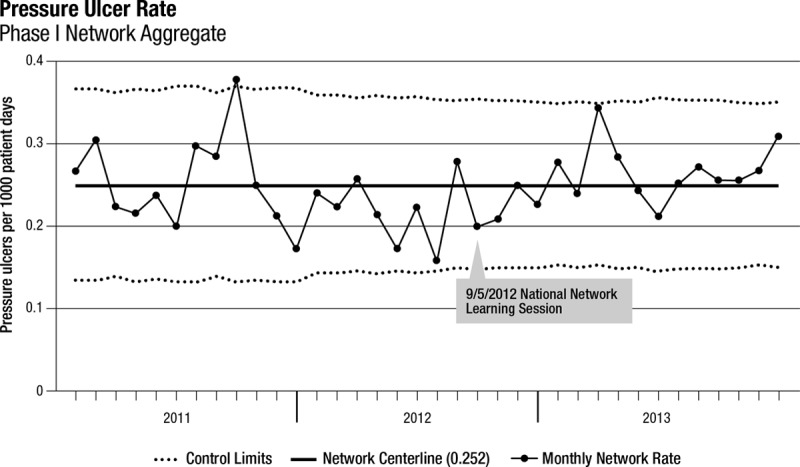
Network aggregate PI rate over time.

In the regression model, there was a 54% increase in the mean rate of stage 2 pressure injuries from the baseline rate compared with the study period (*P* < 0.001; Appendix Table, Supplemental Digital Content 1, http://links.lww.com/PQ9/A0). Further, there was a 40% decrease in the mean rate of stage 3 pressure injuries (*P* = 0.01) compared with baseline and a 58% reduction in the mean rate of stage 4 pressure injuries (*P* = 0.04) compared with baseline. The mean rate of deep tissue injuries rose by 58% from baseline to study period (*P* < 0.001), and the mean rate of unstageable PI had no significant change from baseline compared with study period (*P* = 0.15). Finally, the mean total PI rate was higher during the study period by 19% (*P* = 0.03) compared with the baseline period (Fig. 4).

### Recommended Bundle and PI Rates (Phase 1 and 2 Hospitals)

There were 64 respondents (82%) to the survey representing 64 unique phase 1 and 2 hospitals. We removed 4 hospitals from analysis because they did not follow the operational definition of pressure injuries, and we removed 5 hospitals because they did not submit bundle reliability data. The final analysis included 54 hospitals.

**Fig. 5.**
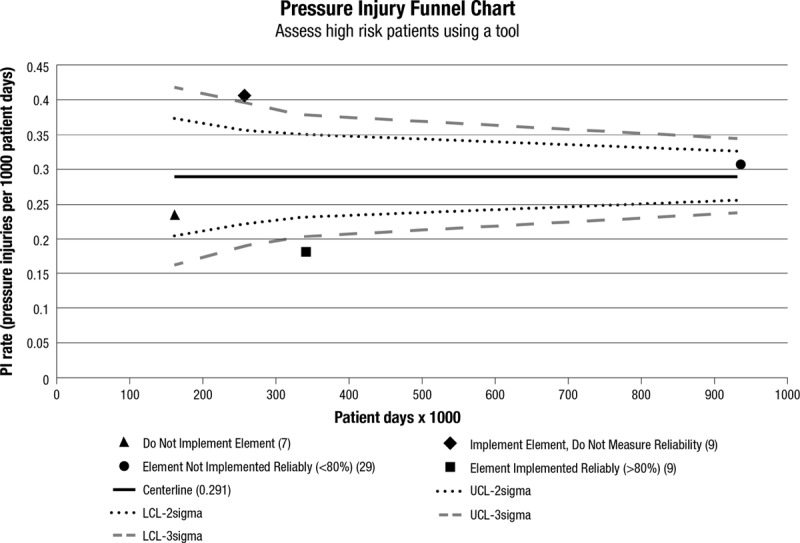
Funnel chart for phase 1 and 2 hospitals (n = 54). This bundle element is “using a tool to assess skin for high risk of pressure injury occurrence.” This funnel chart depicts the relationship between high bundle compliance and PI rates. (All other prevention bundle aspects/elements funnel charts are depicted in Appendix Figs. 1–7, Supplemental Digital Content 2, http://links.lww.com/PQ9/A1.)

In the funnel chart analysis, the cohort that adopted each bundle element, measured compliance, and achieved 80% bundle compliance (reliability) had the lowest PI rates (special cause: below 3-sigma lower control limit) when compared with all hospitals (Fig. 5, Appendix Figs. 1–7, Supplemental Digital Content 2, http://links.lww.com/PQ9/A1).

For example, for the bundle element “High-Risk Patient Skin Assessment,” survey results indicated that 7 hospitals did not implement this bundle element and 9 hospitals implemented this component but did not measure bundle compliance (reliability). Of the remaining 38 hospitals that implemented this bundle element and measured bundle compliance (reliability), 29 did not achieve greater than 80% reliability and 9 did achieve greater than 80% reliability. The rate of pressure injuries for those hospitals that achieved high reliability was below the 3-sigma lower control limit (Fig. 5). We achieved similar results across all 5 bundle elements. In other words, high-reliability bundle compliance correlated with lower PI rates and met special cause criteria (ie, below the 3-sigma lower control limit; Appendix Figs. 1–7, Supplemental Digital Content 2, http://links.lww.com/PQ9/A1).

There was no consistent relationship regarding PI rates for hospitals in the other 3 groups (ie, implemented but did not measure reliability, implemented but did not achieve 80% reliability, or did not implement the bundle element). For example, for the bundle element “Skin Assessment,” hospitals that did not implement the bundle had lower pressure ulcer rates than hospitals that implemented the bundle and did not measure or did not achieve 80% reliability.

## DISCUSSION

The SPS mission is to “work together to eliminate serious harm across all children’s hospitals.” The PI collaborative had 2 objectives: (1) to increase the detection of pressure injuries through active surveillance and (2) to reduce the number of serious pressure injuries (stage 3, stage 4, unstageable pressure injuries, and deep tissue injuries). We found a statistically significant increase in stage 2 pressure injuries and deep tissue injuries and concurrent statistically significant decreases in stage 3 and stage 4 pressure injuries. We believe both these trends reflect improved detection of pressure injuries (better surveillance) at an earlier stage of injury, thus reducing progression to more severe injuries. Also, we found that hospitals that reported greater than 80% bundle reliability also reported fewer pressure injuries.

To our knowledge, this is the first study demonstrating the effectiveness of an evidence-based PI prevention bundle when used in conjunction with active surveillance to detect pressure injuries at the earliest possible stage of injury. This study also adds to the growing body of literature that supports the use of bundles to prevent hospital-acquired conditions.^20^ We studied the PI prevention bundle in a network that used Institute for Healthcare’s Breakthrough Series methodology to share improvement strategies. This methodology allows rapid spread of best practices among participating hospitals.

There is little literature on bundle use in pressure ulcer prevention. As in other bundles, such as the well-studied central line-associated bloodstream infection prevention bundles, this study showed an association between improved compliance with bundle elements and improved clinical outcomes.^18^ We did not find a linear relationship between compliance with individual bundle elements and clinical outcomes. Theoretically, there is a threshold of bundle compliance that leads to improved outcomes. This threshold is traditionally measured at 80% compliance.^16^

To completely eliminate pressure injuries in children, further work is needed. Some hospitals are not reliably following the PI bundle. Our network does not include children who may develop pressure injuries in community hospitals or in the home setting. Also, none of the hospitals has eliminated all pressure injuries, indicating that additional research and quality improvement work are needed to improve the science of PI prevention.

An important aspect of PI reduction in children that was not specifically addressed by this study is the predilection for certain types of devices to cause pressure injuries. The bundle element, Device Rotation, partly addresses this issue, but, in reality, specific strategies are needed for each type of medical device. For example, in our experience, patients with BiPAP masks are at high risk for pressure injuries on the bridge of the nose, but BiPAP masks are not amenable to device rotation. Further study of device-related pressure injuries and improvements in equipment and technology, especially sized and geared for children, are needed before even greater PI reduction can occur.

Although we conducted this project as an improvement collaborative with guiding principles such as “act with a sense of urgency” and “all teach, all learn,” there are certain limitations with this approach. For example, there were multiple interventions including both active surveillance and bundle implementation, with variable penetrance. Although this approach has been very successful in implementing change and demonstrating compelling results, there are certain limitations for research as compared with a randomized controlled study. First, we are not able to make conclusions regarding PI rates at individual hospitals as it relates to their bundle compliance. Rather, this study demonstrates a change in the rates of pressure injuries for the network as a whole over time. Additionally, there was variation among hospitals regarding which bundle elements were adopted, how bundle compliance was measured (ie, direct observation versus documentation in the medical record), and the number of bundle observations. As a result of the analytical methods described in this article, we can conclude that the 5 bundle elements are critical when applied as a bundle, but we are not able to attribute specific results to specific bundle elements.

## CONCLUSIONS

Using a strategy that involves active team-based surveillance for PI detection and implementation of a 5-component PI prevention bundle, injury rates for stage 3 and 4 pressure injuries were significantly reduced and improved detection of stage 2 pressure injuries and deep tissue injuries occurred. We believe that this approach can be emulated and implemented in all hospitals caring for children to reduce the harm associated with hospital-acquired pressure injuries.

## DISCLOSURE

The authors have no financial interest to declare in relation to the content of this article. This study was supported by departmental resources.

## ACKNOWLEDGMENT

The authors would like to acknowledge Trish Burdette and Cindy Henderson in Atlanta and Brenda Ruth and Michelle Miller at Nationwide for their assistance with this work.

## Supplementary Material
