# Supplementary material for: Impact of a Pressure Injury Prevention Bundle in the Solutions for Patient Safety Network
Source: Pediatr Qual Saf. 2017 Feb 16;2(2):e013. doi: 10.1097/pq9.0000000000000013 (PMC6132915; doi:10.1097/pq9.0000000000000013)
Supplement: Supplementary file 1 [file pqs-2-e013-s001.docx]

| **Appendix table**. Estimate of Rate Change in Pressure Injuries with Mixed Effects Negative Binomial Model | | | | | |
| --- | --- | --- | --- | --- | --- |
| Pressure Injury Type | Mixed Effects Negative Binomial Model^#^ | | | | |
|  | Implementation period (2012) | |  | Study period (2013) | |
|  | Rate change (ref=2011 Baseline) | p-value |  | Rate change (ref=2011 Baseline) | p-value |
| Stage 2 | +35% | <0.001 |  | +54% | <0.001 |
| Stage 3 | -16% | 0.36 |  | -40% | 0.01 |
| Stage 4 | -55% | 0.06 |  | -58% | 0.04 |
| Deep Tissue Injuries | +10% | 0.41 |  | +58% | <0.001 |
| Unstageable | -10% | 0.41 |  | +18% | 0.15 |
| Total* | -6% | 0.44 |  | +19% | 0.03 |
| # Baseline period (2011) was used as reference phase when calculating rate ratio and rate change | | | | | |
| * Total pressure injury includes stage 3, stage 4, Deep tissue injuries and unstageable pressure injuries | | | | | |
