# Supplementary material for: Impact of a Pressure Injury Prevention Bundle in the Solutions for Patient Safety Network
Source: Pediatr Qual Saf. 2017 Feb 16;2(2):e013. doi: 10.1097/pq9.0000000000000013 (PMC6132915; doi:10.1097/pq9.0000000000000013)
Supplement: Supplementary file 2 [file pqs-2-e013-s002.pdf]

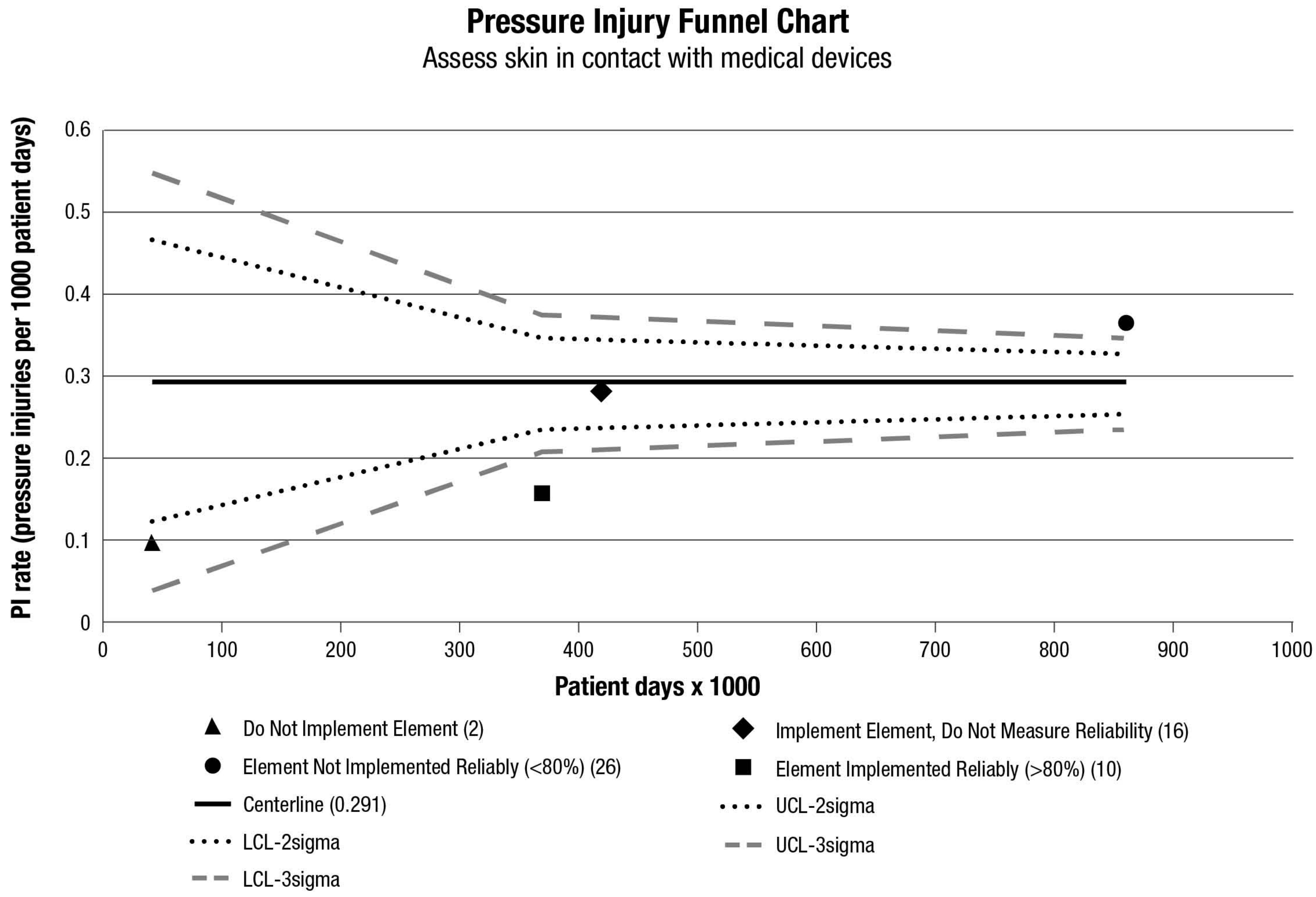

Pressure Injury Funnel Chart
Assess skin in contact with medical devices
PI rate (pressure injuries per 1000 patient days)
0.6
0.5
0.4
0.3
0.2
0.1
0
0
100
200
300
400
500
600
700
800
900
1000
Patient days x 1000
Do Not Implement Element (2)
Implement Element, Do Not Measure Reliability (16)
Element Not Implemented Reliably (<80%) (26)
Element Implemented Reliably (>80%) (10)
Centerline (0.291)
UCL-2sigma
LCL-2sigma
UCL-3sigma
LCL-3sigma

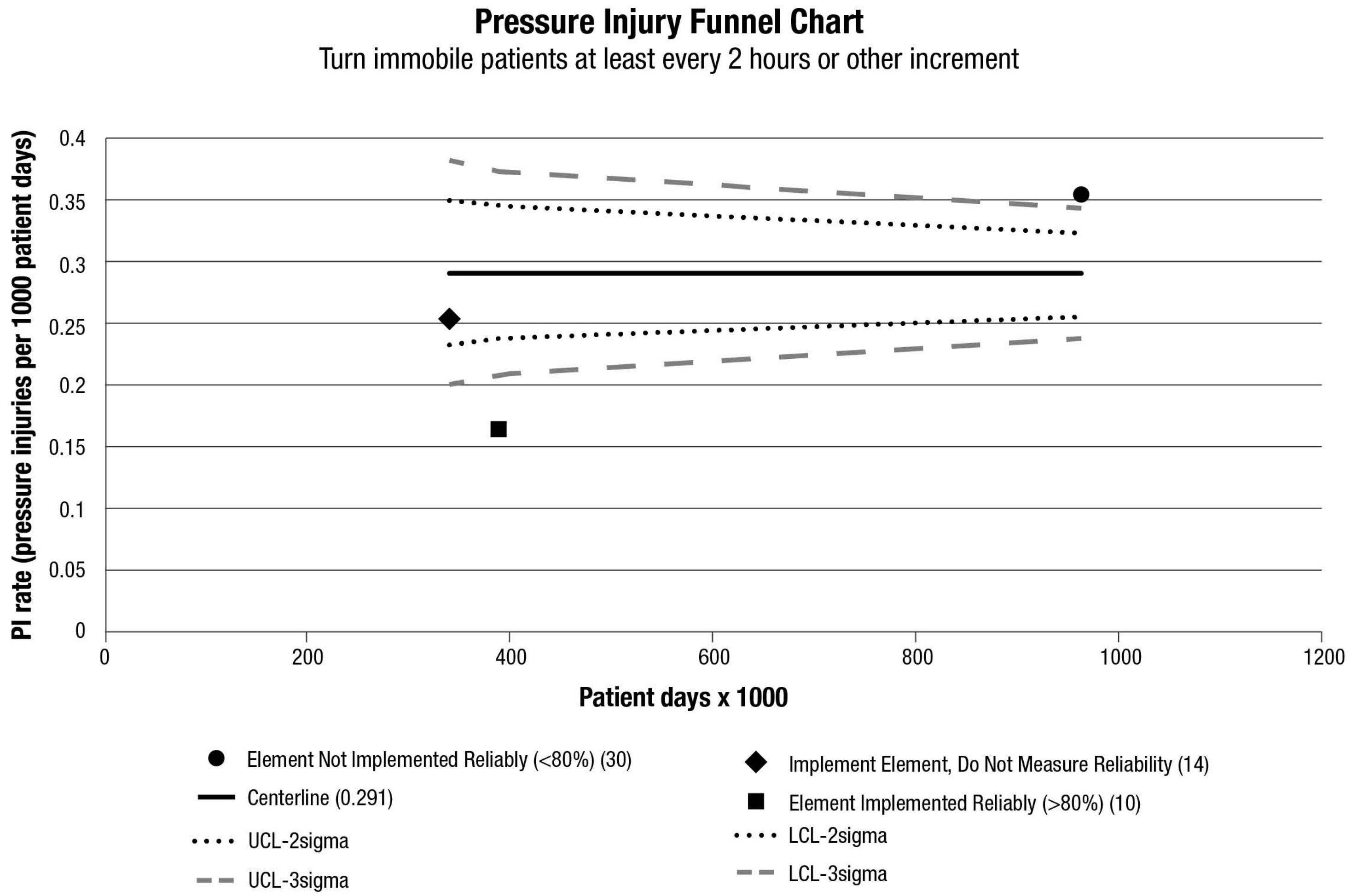

Pressure Injury Funnel Chart
Turn immobile patients at least every 2 hours or other increment
PI rate (pressure injuries per 1000 patient days)
0.4
0.35
0.3
0.25
0.2
0.15
0.1
0.05
0
0
200
400
600
800
1000
1200
Patient days x 1000
Element Not Implemented Reliably (<80%) (30)
Centerline (0.291)
UCL-2sigma
UCL-3sigma
Implement Element, Do Not Measure Reliability (14)
Element Implemented Reliably (>80%) (10)
LCL-2sigma
LCL-3sigma

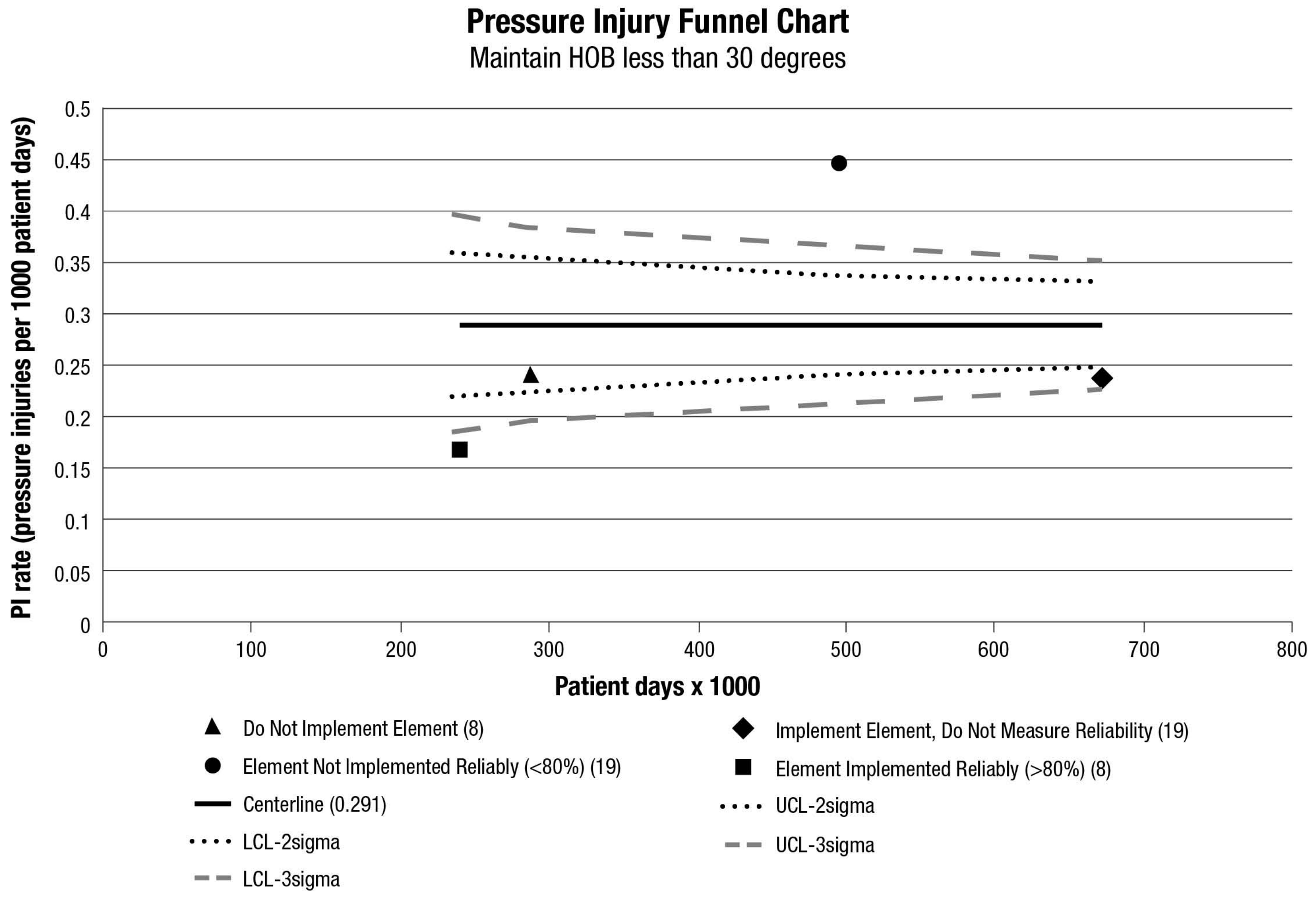
Pressure Injury Funnel Chart
Maintain HOB less than 30 degrees
PI rate (pressure injuries per 1000 patient days)
0.5
0.45
0.4
0.35
0.3
0.25
0.2
0.15
0.1
0.05
0
0
100
200
300
400
500
600
700
800
Patient days x 1000
Do Not Implement Element (8)
Implement Element, Do Not Measure Reliability (19)
Element Not Implemented Reliably (<80%) (19)
Element Implemented Reliably (>80%) (8)
Centerline (0.291)
UCL-2sigma
LCL-2sigma
UCL-3sigma
LCL-3sigma

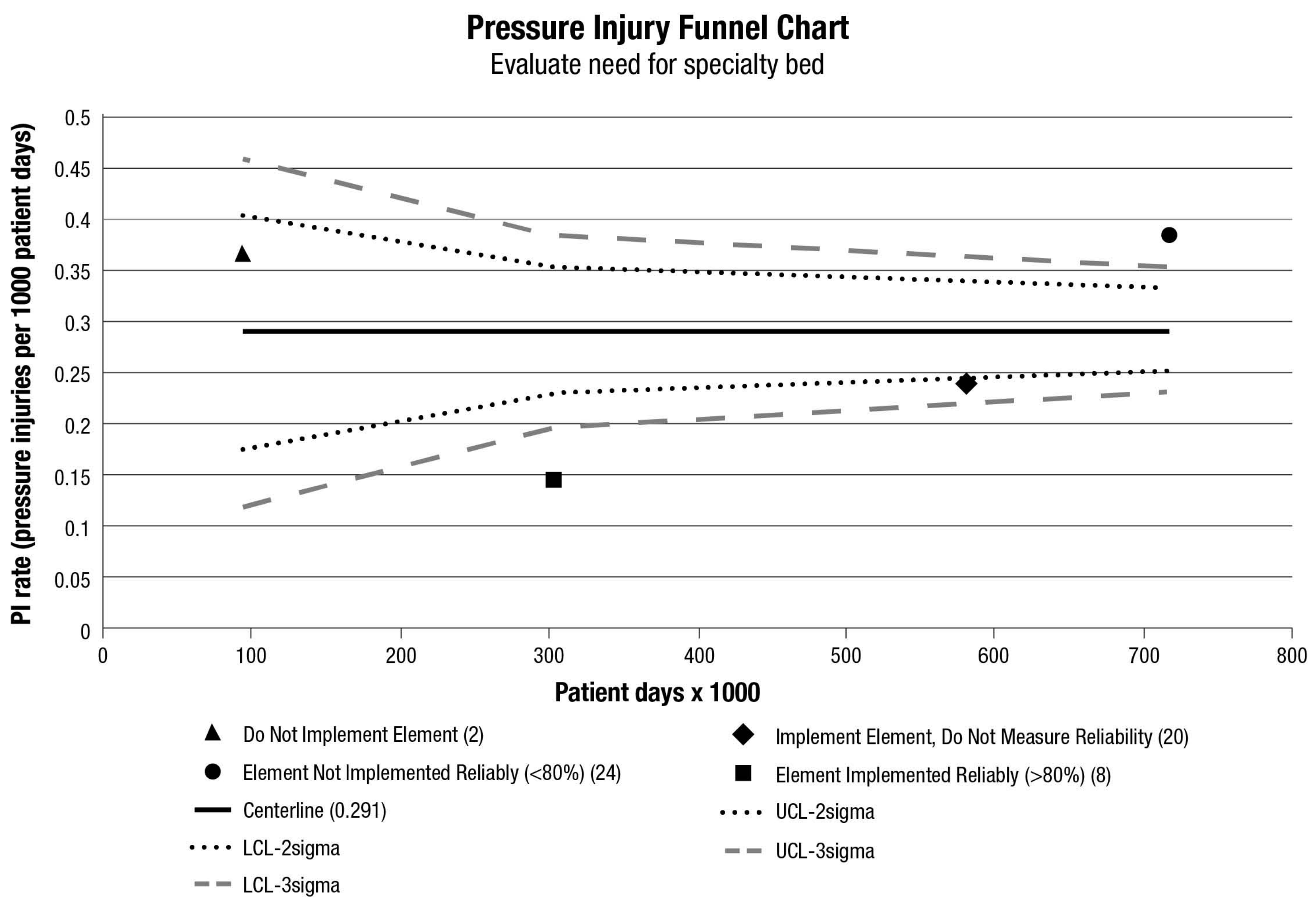

Pressure Injury Funnel Chart
Evaluate need for specialty bed
PI rate (pressure injuries per 1000 patient days)
0.5
0.45
0.4
0.35
0.3
0.25
0.2
0.15
0.1
0.05
0
0
100
200
300
400
500
600
700
800
Patient days x 1000
Do Not Implement Element (2)
Implement Element, Do Not Measure Reliability (20)
Element Not Implemented Reliably (<80%) (24)
Element Implemented Reliably (>80%) (8)
Centerline (0.291)
UCL-2sigma
LCL-2sigma
UCL-3sigma
LCL-3sigma

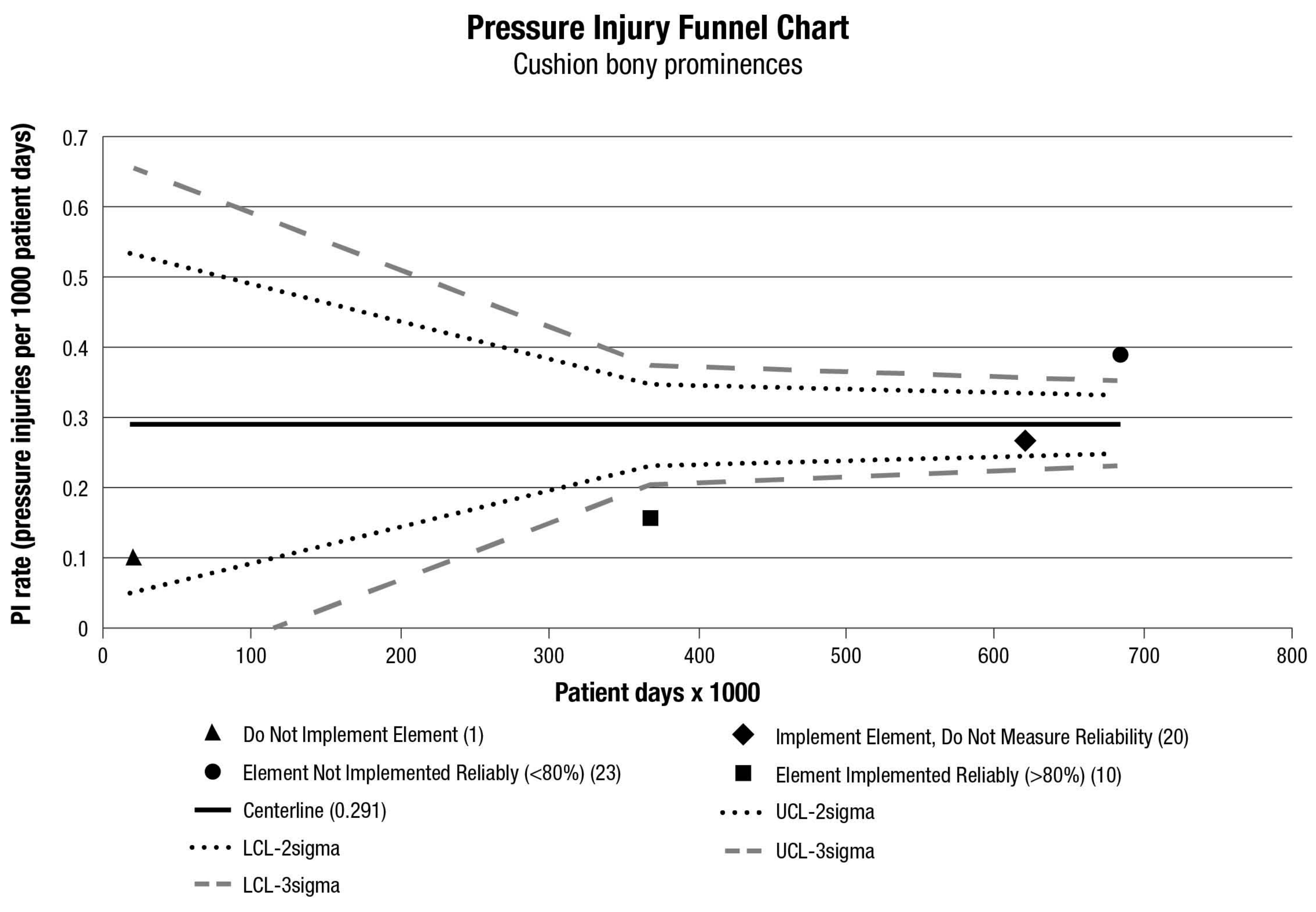

Pressure Injury Funnel Chart
Cushion bony prominences
PI rate (pressure injuries per 1000 patient days)
0.7
0.6
0.5
0.4
0.3
0.2
0.1
0
0
100
200
300
400
500
600
700
800
Patient days x 1000
Do Not Implement Element (1)
Implement Element, Do Not Measure Reliability (20)
Element Not Implemented Reliably (<80%) (23)
Element Implemented Reliably (>80%) (10)
Centerline (0.291)
UCL-2sigma
LCL-2sigma
UCL-3sigma
LCL-3sigma

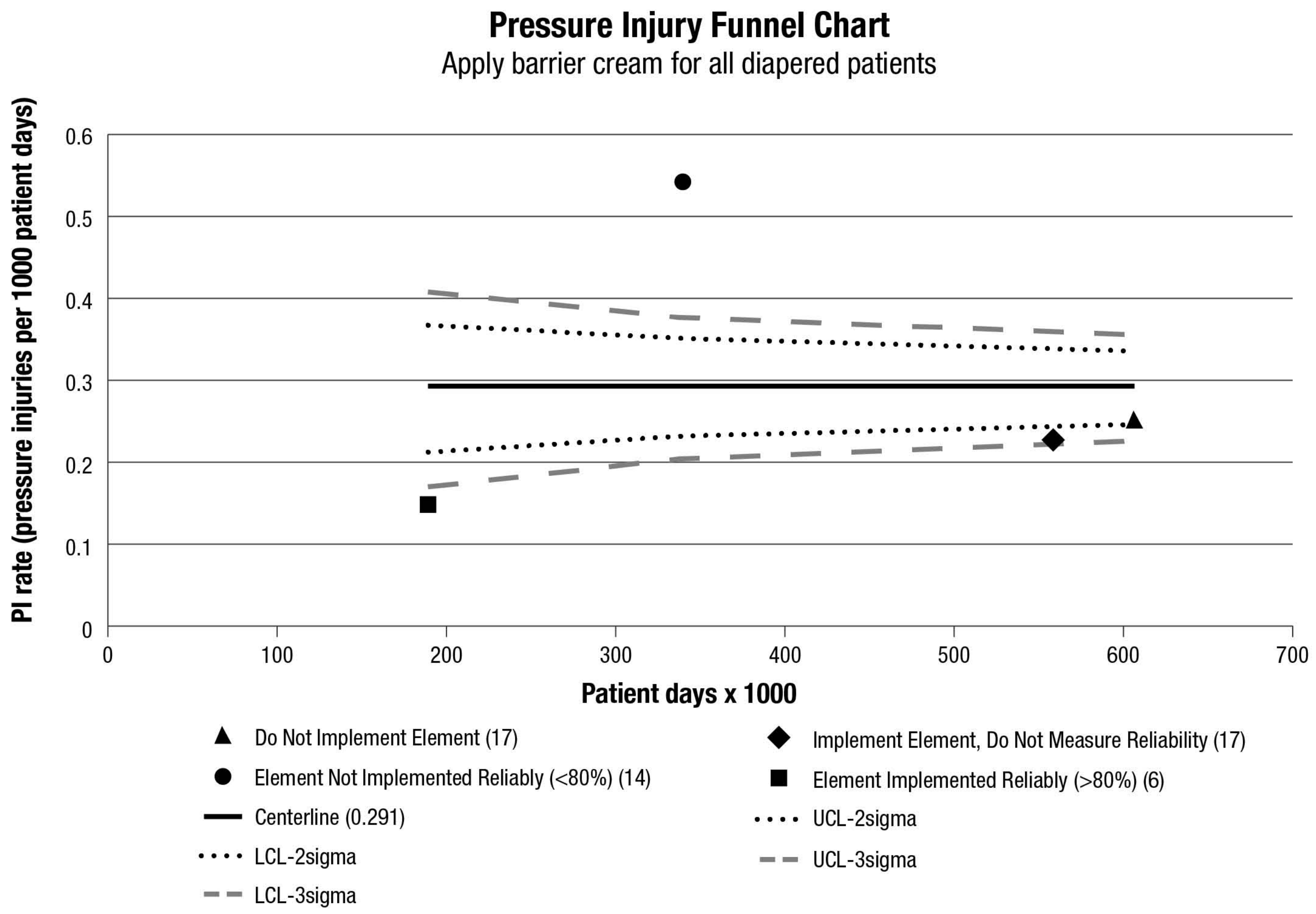
Pressure Injury Funnel Chart
Apply barrier cream for all diapered patients
PI rate (pressure injuries per 1000 patient days)
0.6
0.5
0.4
0.3
0.2
0.1
0
0
100
200
300
400
500
600
700
Patient days x 1000
Do Not Implement Element (17)
Implement Element, Do Not Measure Reliability (17)
Element Not Implemented Reliably (<80%) (14)
Element Implemented Reliably (>80%) (6)
Centerline (0.291)
UCL-2sigma
LCL-2sigma
UCL-3sigma
LCL-3sigma

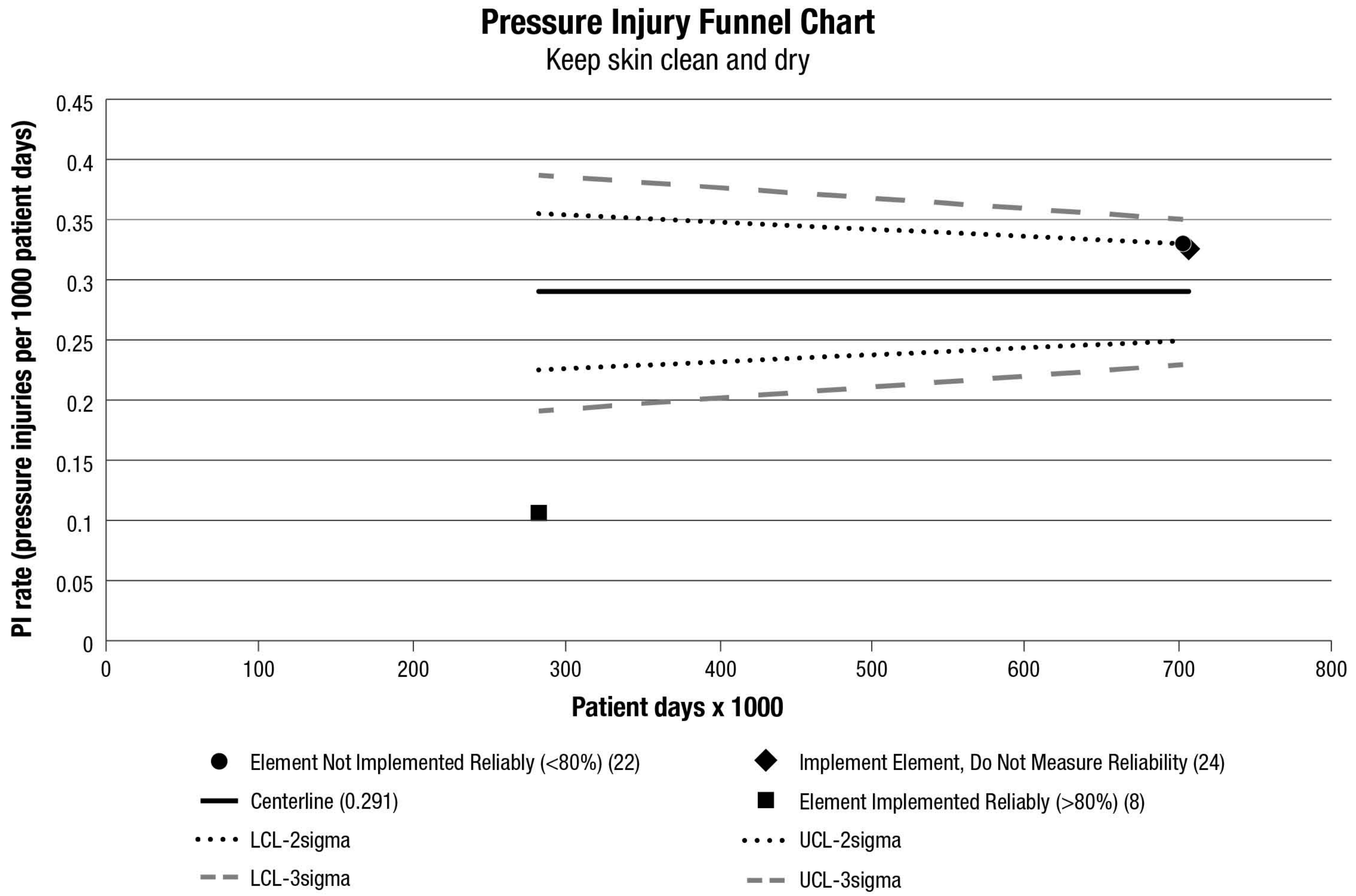
Pressure Injury Funnel Chart
Keep skin clean and dry
PI rate (pressure injuries per 1000 patient days)
0.45
0.4
0.35
0.3
0.25
0.2
0.15
0.1
0.05
0
0
100
200
300
400
500
600
700
800
Patient days x 1000
Element Not Implemented Reliably (<80%) (22)
Implement Element, Do Not Measure Reliability (24)
Centerline (0.291)
Element Implemented Reliably (>80%) (8)
LCL-2sigma
UCL-2sigma
LCL-3sigma
UCL-3sigma
